# *CORR* Insights^®^: Transcriptional Profiling Identifies the Signaling Axes of IGF and Transforming Growth Factor-β as Involved in the Pathogenesis of Osteosarcoma

**DOI:** 10.1007/s11999-015-4620-3

**Published:** 2015-11-12

**Authors:** Elisabeth F. P. Peterse, Judith V. M. G. Bovée

**Affiliations:** Department of Pathology, Leiden University Medical Center, Albinusdreef 2, 2333 ZA Leiden, The Netherlands

## Where Are We Now?

Targeted therapeutics are needed to improve the prognosis of patients with osteosarcoma. Since the introduction of chemotherapy in the 1980s, 5-year survival for the often young patients with osteosarcoma has plateaued at about 50% to 60%. For patients with metastases, the outcomes remains particularly poor. By performing a serial analysis of a gene expression experiment comparing osteosarcomas with normal osteoblasts and mesenchymal stem cells, the authors identified the insulin-like growth factor (IGF) pathway and the transforming growth factor-β (TGF-β) pathway as possible targets for therapy. Since the effects of stimulation or inhibition of the TGF-β pathway on osteosarcoma cell proliferation is debatable [9], and the developed TGF- β pathway inhibiting agents elicit unwanted effects [13], we will focus on the possibility of targeting the IGF pathway in osteosarcoma.

The current paper emphasizes that the IGF pathway plays an important role in osteosarcoma pathogenesis, which is supported by other preclinical studies showing reduced proliferation in the majority of osteosarcoma cell lines [7] and xenografts [6] upon IGF 1 receptor (IGF-1R) inhibition. In addition, the peak incidence of osteosarcoma correlates with the increased levels of growth hormone and IGF ligands in puberty, and it has been described that the expression of several IGF pathway members (IGF-1R, IGF-1, growth arrest-specific 6, and IGF binding proteins 4 [IGFBP4]) correlates with osteosarcoma prognosis [4, 7]. However, clinical trials demonstrate that only a small subset of osteosarcoma patients respond to IGF-1R antibodies [3]. This illustrates the general trend with IGF-1R inhibitors. Although preclinical models have shown promising results, evidence for their efficacy in large-scale randomized controlled trials is lacking. As a consequence, pharmaceutical companies have discontinued the production of all IGF pathway targeting agents.

## Where Do We Need To Go?

The current paper emphasizes the importance of IGF signaling in the development of osteosarcomas. Despite the fact that only a subset of patients with osteosarcoma are sensitive to IGF-1R inhibition, the strong preclinical rationale deserves further clinical and translational exploration. Predictive biomarkers to identify the subset of patients that will benefit from IGF-directed therapy should be identified. The most obvious biomarker would be expression of the IGF-1R, but a Phase 2 trial with Cixutumumab (IGF-1R antibody) and Temsirolimus (mTOR inhibitor) in patients with bone and soft-tissue sarcomas showed that IGF-1R expression does not correlate with therapeutic response [11]. However, the search for biomarkers is ongoing, and several other biomarkers have been proposed, such as mutated Kirsten rat sarcoma viral oncogene homolog (KRAS) and phosphorylated extracellular signal-regulated kinases (ERK) [5].

Besides the identification of predictive biomarkers, the efficacy of alternative methods to target the IGF pathway should be explored, such as tyrosine kinase inhibitors that target both the IGF-1R and the insulin receptor, given its crossreactivity. Once in vitro studies identify the best approach to target the IGF pathways, and once biomarkers have been identified, the next step would be small clinical trials to reevaluate the efficacy of IGF pathway inhibitors in patients with osteosarcoma. These clinical trials must incorporate a strong translational research program. Therefore, we should convince pharmaceutical companies to resume the production of IGF pathway inhibitors, especially the IGF-1R/insulin receptor dual inhibitors.

## How Do We Get There?

One clinical trial with Linsitinib (a dual IGF-1R insulin receptor inhibitor) is currently being performed in Ewing sarcoma, where IGFBP3 is downregulated as a consequence of the Ewing sarcoma breakpoint region 1-Friend leukemia virus integration 1 fusion gene (EWSR1-FLI-1) [10] resulting in overactivity of the IGF pathway. Interestingly, IGF-1R inhibition seems promising in a subset of patients with Ewing sarcoma [3]. This EuroSARC study, led by Prof. Dr. Hassan from the University of Oxford, aims to identify predictive biomarkers by incorporating a strong translational research program in this clinical trial. The biomarkers that will be identified in the EuroSARC study should be tested in other tumors including osteosarcoma, and as mentioned above, new trials for osteosarcoma, similar to this trial in Ewing sarcoma, should be conducted. The clinical trials that have been performed in osteosarcoma thus far have two major limitations. First, all the studies involved IGF-1R monoclonal antibodies. It is known that signaling via the insulin receptor can circumvent IGF-1R inhibition [2]. Indeed, it was shown in osteosarcoma cells that dual inhibition of IGF-1R and insulin receptor is more effective than IGF-1R inhibition alone [1]. Therefore, results from clinical studies with dual inhibitors are warranted. Second, in the clinical trials conducted so far, IGF-1R-targeting antibodies were administered as a monotherapy to patients that failed complete remission upon chemotherapy. IGF-1R inhibitors should be tested in combination with chemotherapeutic agents, as it has been described that the ligand IGF-2 is upregulated in patients in response to chemotherapy [12] and that in osteosarcoma cell lines IGF-1R inhibition can enhance the effect of doxorubicin and radiotherapy [8].

The current paper strengthens the view that the IGF pathway can be an effective target for therapy in osteosarcoma, thereby emphasizing the need for the industrial facilitation of clinical trials with IGF-1R/insulin receptor dual inhibitors combined with chemotherapy, in which a strong translational program is embedded for predictive biomarker identification in osteosarcoma patients.
